# Corticotropin Releasing Factor promotes breast cancer cell motility and invasiveness

**DOI:** 10.1186/1476-4598-8-30

**Published:** 2009-06-02

**Authors:** Ariadne Androulidaki, Erini Dermitzaki, Maria Venihaki, Effie Karagianni, Olga Rassouli, Erini Andreakou, Christos Stournaras, Andrew N Margioris, Christos Tsatsanis

**Affiliations:** 1 Department of Clinical Chemistry, School of Medicine, University of Crete, Heraklion 71003, Crete, Greece; 2 Department of Biochemistry, School of Medicine, University of Crete, Heraklion 71003, Crete, Greece

## Abstract

**Introduction:**

Cancer cells secrete bioactive peptides that act in an autocrine or paracrine fashion affecting tumor growth and metastasis. Corticotropin-releasing factor (CRF), a hypothalamic neuropeptide that controls the response to stress, has been detected in breast cancer tissues and cell lines. CRF can affect breast cancer cells in an autocrine or paracrine manner via its production from innervating sympathetic neurons or immune cells.

**Methods:**

In the present study we report our findings regarding the impact of CRF on breast cancer cell motility and invasiveness. For this purpose we used the MCF7 breast cancer cell line and evaluated the effect of CRF on motility and invasiveness using the wound-healing and boyden-chamber assays. In addition, we measured the effect of CRF on molecules that mediate motility by western blot, immunofluorescence, ELISA and RT-PCR.

**Results:**

Our findings show that: 1. CRF transiently inhibited the apoptosis of MCF7 cells. 2. CRF enhanced MCF7 cell motility in a wound healing assay and their invasiveness through extracellular matrix. 3. CRF increased actin polymerization, phosphorylation of Focal Adhesion Kinase (FAK), providing a potential mechanism for the observed induction of MCF7 motility. 4. CRF induced the expression of Cox-1 but not Cox-2 in MCF7 cells as well as the production of prostaglandins, factors known to promote invasiveness and metastasis.

**Conclusion:**

Overall, our data suggest that CRF stimulates cell motility and invasiveness of MCF7 cells most probably via induction of FAK phosphorylation and actin filament reorganization and production of prostaglandins via Cox1. Based on these findings we postulate that the stress neuropeptide CRF present in the vicinity of tumors (either produced locally by the tumor cells themselves or by nearby normal cells or secreted from the innervations of surrounding tissues) may play an important role on breast tumor growth and metastatic capacity, providing a potential link between stress and tumor progression.

## Introduction

Neuropeptides and their receptors are present in the tumor microenvironment affecting cancer progression [1-4]. Neuropeptides are known to be produced either from the tumor cells themselves or by nearby located non-tumor cells, such as stroma, immune cells or by innervating autonomic neurons. Corticotropin-releasing factor (CRF) is the major hypothalamic mediator of the response to stress. CRF is also a well-known homeostatic paracrine modulator in the periphery. CRF peptides and their receptors are also expressed in several types of tumors [5-8].

The neuropeptide CRF and its family members Urocortin (UCN)1, UCN2 and UCN3 act via two receptors, CRF1 and CRF2, subtypes of which are differentially expressed in the central nervous system (CNS) and a multitude of peripheral tissues [9,10]. Apart of the well-characterized role of CRF in the homeostatic response to stress, several actions in peripheral tissues have also been described. The CRF system has been implicated in the physiology of the cardiovascular, reproductive and gastrointestinal systems [11-13]. Moreover, CRF peptides and their receptors are also present in the immune system and possess immunomodulatory properties [14-18].

Peptides of the CRF family and their receptors have been detected in various tumors. Several neuroendocrine tumor cell lines such as the PC12 pheochromocytoma, Y79 retinoblastoma, IMR-32 and SH-SY5Y neuroblastoma, AtT-20 pituitary carcinoma and NCI-H82 small cell lung cancer cell lines express CRF and the CRF1 receptor [19-22]. In addition, epithelial tumors and epithelial tumor cell lines express CRF receptors. CRF1 receptors have been detected in the MCF7 breast cancer cell line, while CRF immunoreactivity has been reported in surgical breast cancer specimen, suggesting a role for the CRF/CRF-receptor system in breast cancer [23]. CRF and its receptors are also expressed in human melanomas and in melanoma cell lines [6,7]. It should be noted here that CRF is constantly present in the microenvironment of tumors produced by nearby cells including endothelial cells [24] and immune cells [14] and by the local neuronal innervations [25].

A number of reports support both a tumor-promoting and a tumor-inhibitory effect of CRF peptides. Thus, in the endometrial adenocarcinoma cell line Ishikawa UCN and CRF inhibit cell proliferation via CRF1 [5]. UCN was also shown to inhibit the proliferation of melanoma cells both in vitro and in vivo, through CRF1 [26]. In the human breast cancer cell line MCF7, CRF inhibits estrogen-induced proliferation via CRF1 [23]. Moreover, CRF and CRF related peptides, sauvagine and UCN, inhibit the proliferation of human HaCaT keratinocytes via CRF1 [27]. In addition, CRF has been found to induce the expression of Fas ligand and apoptosis in the rat PC12 pheochromocytoma cell line also via CRF1 [28]. In contrast, in the Y79 retinoblastoma cell line CRF suppresses apoptosis via downregulation of pro-caspase 3 cleavage and activation [29]. It should be mentioned here that the tumor-promoting properties for CRF can be supported by the fact that CRF induces Fas ligand production in ovarian cancers, an effect resulting in cytotoxic T-cell apoptosis and local immunosuppression [8]. Interestingly, ligands of the other CRF receptor, the CRF2, have been found to suppress tumor growth while the expression of the CRF2-specific endogenous ligand UCN2 in tumors results in reduced angiogenesis and suppression of tumor growth [30].

Even though expression of CRF and its receptors has been described in different types of cancer cells, the role of these peptides in tumor growth and metastasis has not been elucidated. The aim of this work was to study the role of CRF in breast cancer cell homeostasis, motility and invasiveness. For this purpose we utilized the MCF7 breast cancer cell line and found that while CRF affected apoptosis it also promoted cell motility and invasiveness, supporting a tumor-promoting role for CRF and CRF1 signals.

## Methods

### Cell culture

The human breast cancer cell line MCF7 was cultured in Dulbecco's Modified Eagle Medium (DMEM) supplemented with 10% fetal calf serum (FCS) and 1% penicillin/streptomycin (all purchased from Invitrogen, UK), at 37°C in a 5% CO2 humidified atmosphere. Cells were plated at a concentration of 2 × 10^5 ^cells/ml until next day when they had reached at approximately 80% confluence. The medium was replaced with serum-free one and cells where stimulated with CRF (Tocris, UK) at a concentration of 10^-8 ^M for different time points. Control cells were treated with the CRF diluent, being 0.1% acetic acid. When antagonists or inhibitors were used, antagonists were administered one hour prior to stimulation with the peptide; a-helical CRF (Sigma, USA) and astressin-2B (kindly provided by Dr. J. Spiess), antagonists of CRF1 and CRF2 respectively, were used at a concentration of 10^-6 ^M.

### RNA isolation and Reverse Transcription-PCR

Total cellular RNA was isolated using Trizol reagent (Invitrogen, UK). Following reverse transcription (Thermoscript RT, Invitrogen, UK), 1 μl of the cDNA product was amplified by PCR (Platinum Taq polymerase, Invitrogen, UK). The primer sets, previously reported to detect the respective CRF receptors in human brain specimen [31], were: CRF1a (272 bp): sense 5'-GGCAGCTAGTGGTTCGGCC-3' and antisense 5'-TCGCAGGCACCGGATGCTC-3'; CRF1b: sense 5'-GGCCAGGCTGCACCCATTG-3'; antisense 5'-TCGCAGGCACCGGATGCTC-3'; CRF2a: sense 5'-ATGGACGCGGCACTGCTCCA-3'; antisense 5'-CACGGCCTCTCCACGAGGG-3'. For CRF2b (342 bp) sense 5'-GGGGCTGGCCAGGGTGTGA-3' and antisense 5'-CACGGCCTCTCCACGAGGG-3'. CRF2c (300 bp): sense 5'-CTGTGCTCAAGCAATCTGCC-3' and antisense 5'-CACGGCCTCTCCACGAGGG-3'. Beta-Actin (214 bp): sense 5'-CCGGCCAGCCAGGTCCAGA-3' and antisense 5'-CAAGGCCAACCGCGAGAAGATG-3'. Products were amplified using the following PCR conditions: denaturation at 95°C for 45 seconds, annealing at 60°C for 45 seconds, extension at 72°C for 45 seconds, for a total of 40 cycles. A sample where no-reverse transcriptase was added during reverse transcription of the RNA (no RT) and another where water was added instead of cDNA, were used as controls. 12 μl of the amplified products were separated on a 2.5% agarose gel and visualized by ethidium bromide staining using the BioRad Molecular Analyst System [32].

### Quantitative measurement of apoptosis

The APOPercentage apoptosis assay (Biocolor Ltd., Belfast, UK) was used to quantify apoptosis, according to manufacturer's instructions. Briefly, cells were plated in flat bottom 96-well plates at a concentration of 15,000 cells/well and the next day medium was replaced by medium free of serum containing CRF at a concentration of 10^-8 ^M, for different time points. One hour before the end of the experiment, 5 μl of the APOPercentage dye was added to each well for one hour. Cells were then washed with PBS and lysed in the Dye Release Reagent. The APOPercentage Apoptosis Assay's dye stained red the apoptotic cells undergoing the membrane flip-flop event, when phosphatidylserine is translocated to the outer leaflet. Apoptosis was quantified after cell lysis by measuring the dye incorporated in apoptotic cells at 550 nm (reference filter 620 m) using an Elisa reader (Biorad, UK).

### Quantitative measurement of cell growth

Cell growth was measured using the yellow tetrazolium MTT assay (Sigma, USA). The yellow tetrazolium MTT is reduced by metabolically active cells, in part by the action of dehydrogenases. Briefly, 15,000 cells were plated in 96 well plates and treated with CRF At the end of the incubation period MTT was added at a concentration of 0.5 mg/ml and incubated for 3 hours. Cells were then lysed by adding 0.04 N HCl in isopropanol and absorbance was measured at 620 nm in an ELISA plate reader (Biorad, UK).

### Wound healing assay

Cells were cultured in 60 mm plates until the surface was completely covered. A small area was then disrupted and a group of cells was destroyed or displaced by scratching a line through the layer with a tip [33]. The culture medium was replaced with serum free medium and cells were stimulated with 10^-8 ^M CRF. The open gap was then inspected microscopically (Leica, Germany) over time as the cells moved in and filled the damaged area. Images were captured at the beginning and at regular time points during cell migration and the cell migration was quantified by measuring the distance with the program Image J  between two certain points on either side of the gap. For proper statistical evaluation, at least three measurements at different points were performed at each image.

### Cell Invasion Assay

The assay was performed in a 96 well invasion plate based on the Boyden chamber principle. The bottom of each well contained an 8 μm pore size polycarbonate membrane coated with a thin layer of Extracellular Matrix (ECM) through which invasive cells migrate to the bottom of the membrane. Invaded cells were dissociated, lysed and quantified by fluorometric analysis using SYBR green, according to the manufacturer's instructions (Chemicon, USA).

### Evaluation of actin reorganization by Confocal Laser Scanning Microscopy

Cells were cultured in 8-well chambers slides (50,000 cells per well). The next day the culture medium was replaced with serum free medium and cells were stimulated with 10^-8 ^M CRF for 1, 3 and 6 hours. At the end of each experiment, cells were harvested, transferred to tubes, washed with PBS and permeabilized by exposure to 3.7% formaldehyde for 10 minutes. Cells were then incubated with acetone for 4 minutes at room temperature, washed with PBS and incubated with 1.5% FCS. Finally, rhodamine-phalloidin was added to the cells at 1:100 dilution in PBS/FCS 1.5% for 30 min in the dark. Subsequently, cells were washed with PBS, analyzed with a confocal laser-scanning module (Leica Lasertechnik, Heidelberg Germany) and images were assessed with the respective software.

### Measurement of monomeric (G) and polymeric (F) actin by Triton X-100 fractionation

The Triton X-100 soluble G-actin and insoluble F-actin-containing fractions of cells exposed to CRF at 10^-8 ^M in serum free medium for 3 and 6 hours were prepared as previously described [34]. The quantification of actin was performed by reference to a standard curve, prepared from muscle actin [34]. The G- and total actin contents were related to the total protein content. Protein concentrations were measured with a commercially available kit (Bio-Rad, UK). A decrease of the triton-soluble (G) to total actin ratio [triton-insoluble (F) + triton-soluble (G)] is indicative of actin polymerization.

### Measurment of FAK phosphorylation (Confocal Laser Scanning Microscopy)

Cells were cultured in 8-well chambers slides (50,000 cells per well). The next day the culture medium was replaced with serum free medium and cells were stimulated with 10^-8 ^M CRF for 3 hours. Cells were harvested, washed with PBS containing NaF and PMSF and incubated with PFA 4% for 10 min. Cells were then washed with PBS and 0.1% Triton X-100 was added for 15 minutes. Then, cells were incubated overnight with a monoclonal antibody against the phosphorylated form of FAK (Cell signaling, USA). Finally, cells were washed with PBS, stained with secondary anti-mouse Ig FITC-conjugated antibody raised in goat, for 1 hour and photographed with a Confocal Laser Scanning Microscope.

### Measurement of total prostaglandin production

2 × 10^5 ^cells were plated in 24 well plates and stimulated with CRF 10^-8 ^for different time points. The supernatants of the cells were collected and stored at -80°C until analyzed. The production of total prostaglandins was measured by the Prostaglandin Screening EIA Kit (Cayman, USA) according to the manufacturer's instructions. The assay is based on the competition between PGs and a PG-acetylcholinesterase conjugate for a limited amount of PG antiserum.

### Western blotting analysis

Western blot analysis of proteins for the detection of tubulin (Chemicon, USA), Cox-1 (Chemicon, USA) and Cox-2 (Santa Cruz, California, USA) was performed as previously described [28]. Briefly, protein content in the lysates was measured by Bradford Assay. SDS-PAGE sample loading buffer was added in 10 μg of protein from each lysate and electrophoresed through a 12% SDS polyacrylamide gel. Protein was transferred to nitrocellulose membranes, using an LKB electroblot transfer system (LKB, Bromma, Sweden). To detect protein levels, membranes were incubated with the appropriate antibodies and then exposed to Kodak X-omat AR films. A PC-based Image Analysis was used to quantify the intensity of each band (Image Analysis INC., Ontario, Canada).

### Statistical analysis

All values were expressed as the average ± Standard Error of data obtained from at least three independent experiments. Comparison between groups was made using the ANOVA test (single factor) and p < 0.05 was the significance level.

## Results

### 1. Expression of CRF receptor subtypes in MCF7 cells

**Figure 1:**
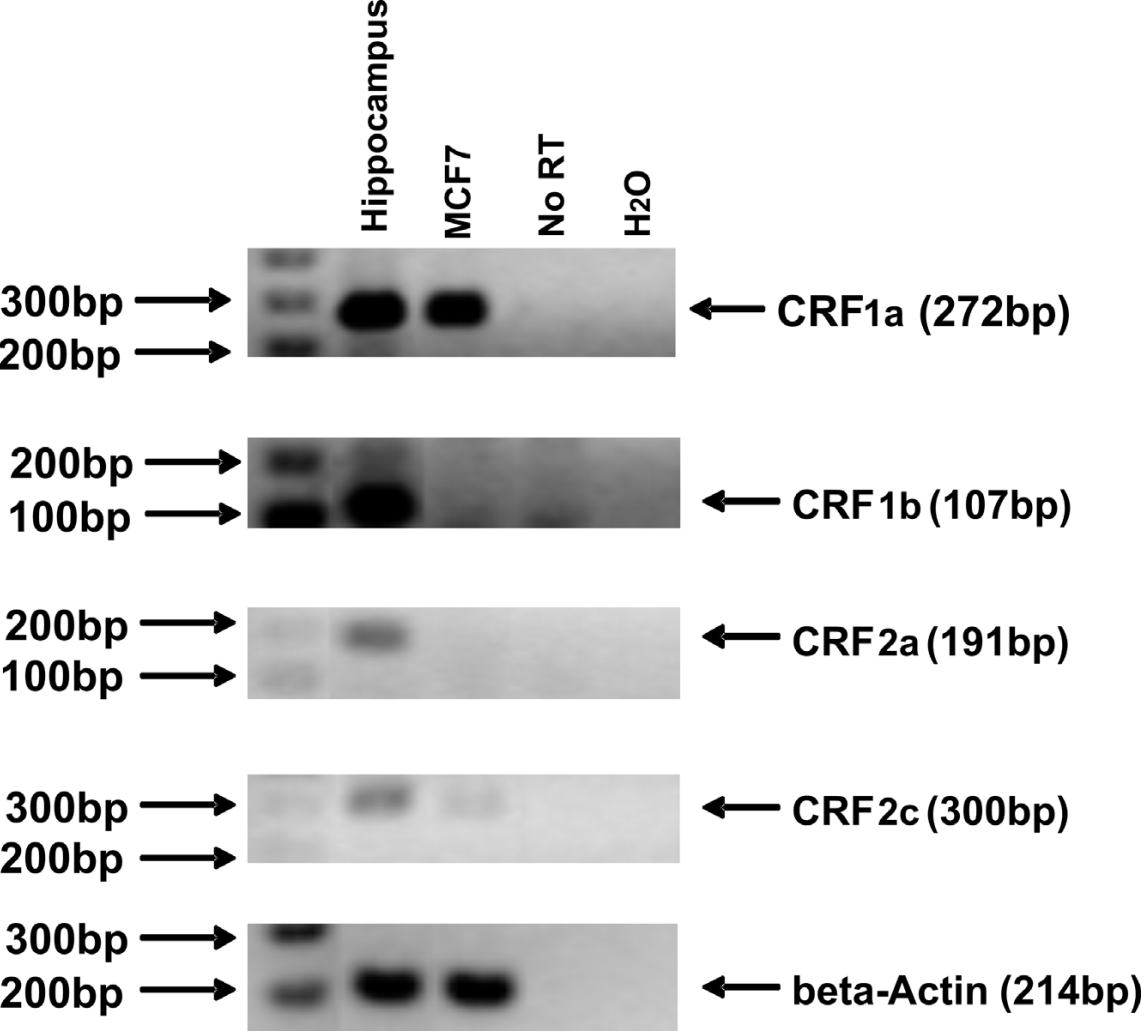
**CRF1a and CRF2c are expressed in MCF7 cells as detected by RT-PCR**. Total RNA was isolated from MCF7 cells and the expression of CRF receptors subtypes CRF1a, CRF1b, CRF2a and CRF2c were detected by semi-quantitative RT-PCR. The levels of beta Actin were also measured as a normalization control. Results are representative of three independent experiments.

To confirm that any biological effect of CRF in MCF7 cells occurred via the characterized CRF receptors we investigated the expression of different CRF receptor subtypes. Expression of CRF1 has been previously reported in MCF7 cells [23]. RNA from MCF7 cells was analyzed for the expression of CRF1a, CRF1b, CRF2a and CRF2c receptor subtypes by RT-PCR. Among these four subtypes, CRF1a mRNA was expressed in high levels while CRF2c mRNA was present at very low levels (Figure 1). The mRNAs of CRF1b, CRF2a were detected in human hippocampus but were not detected in MCF7 cells (Figure 1).

### 2. CRF affects apoptosis of MCF7 cells in a time dependent-manner

**Figure 2:**
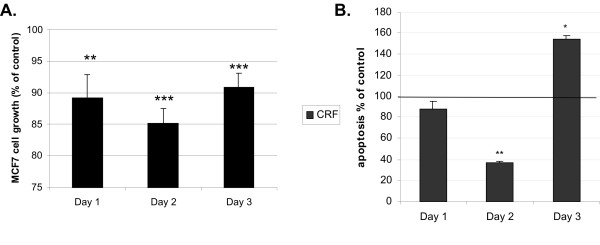
**CRF affected proliferation and apoptosis of MCF7 cells in a time dependent manner**. Cells were stimulated with 10^-8 ^M CRF or vehicle (control) for 24, 48, and 72 hours, and cell growth was measured using the MTT assay (A); apoptosis was measured by the ApoPercentage Assay (B). CRF significantly suppressed proliferation at all time points tested and apoptosis 48 hours after CRF stimulation. Data are expressed as % of control (unstimulated cells). Results represent the average of 4 independent experiments where each condition was assayed in triplicate. *p < 0.05; **p < 0.01 compared to control cells at the same time point.

Evasion of apoptosis is a hallmark of cancer cells and is frequently associated with proliferation and invasiveness [35-38]. It has been previously reported that CRF has anti-proliferative effects on cancer cell lines such as Ishikawa endometrial carcinoma cells and in MCF7 stimulated by estrogens [5,23]. Herein we confirmed that CRF suppresses MCF7 cell proliferation (Figure 2A) and determined its effect on apoptosis by measuring the exposure of phosphatidyl-serine on the cell membrane. MCF7 were treated with CRF at a concentration of 10^-8 ^M for 24, 48, and 72 hours and apoptosis was quantified. Control cells were treated with vehicle (0.1% acetic acid). CRF stimulation significantly protected MCF7 cells from serum deprivation-induced apoptosis becoming evident 48 hours following stimulation (Figure 2B). At later time points apoptosis appeared increased, suggesting a biphasic effect of CRF on apoptosis.

### 3. CRF promotes the motility of MCF7 cells

**Figure 3:**
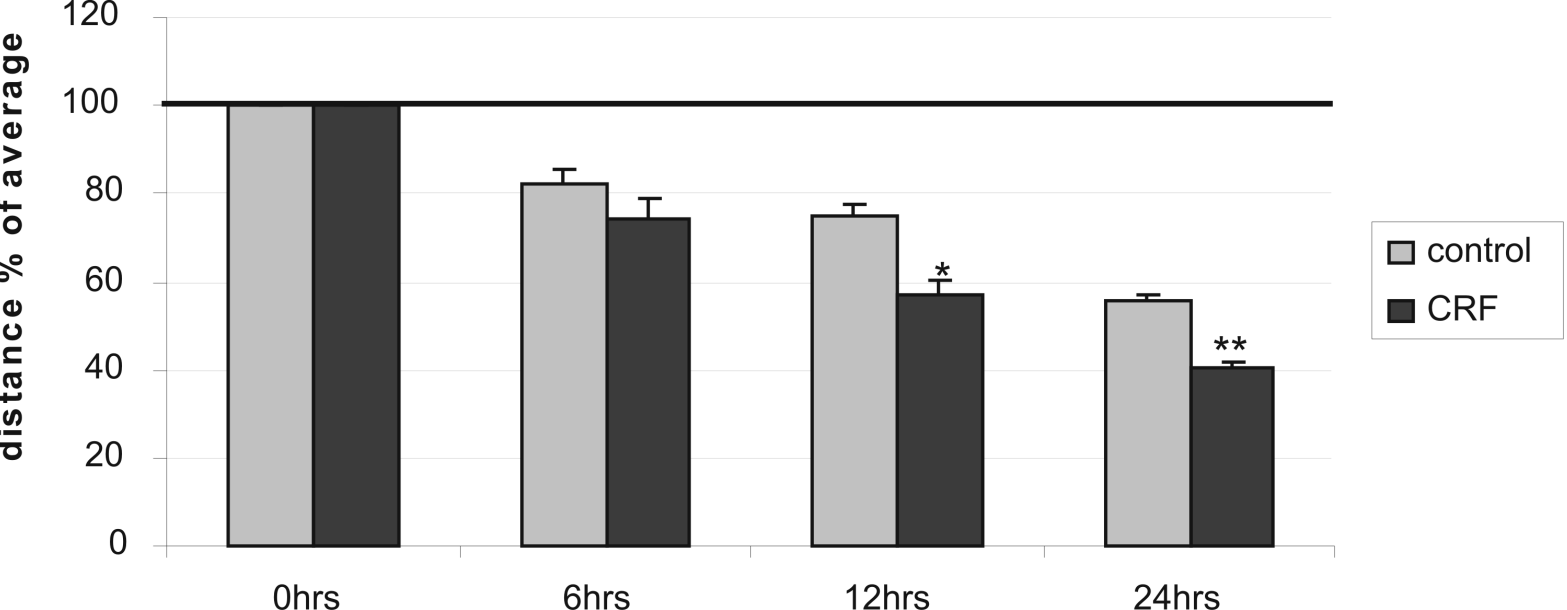
**CRF induces the motility of MCF7 in a wound healing assay**. MCF7 cells were stimulated with 10^-8 ^M CRF or vehicle (control) and photographed at 0, 6, 12 and 24 hours after disruption of a small area of the cell layer. The remigration of the cells was quantified by measuring the distance at 3 at least different positions on each image with the program Image J, and expressed the distance as % of the average. Results represent the average of three independent experiments. *p < 0.05; **p < 0.01 compared to control cells at the same time point.

Increase in cell motility has an impact on the metastatic potential of cancer cells. We, therefore, tested whether CRF could increase motility of MCF7 cells, a cell line with low metastatic potential. To this end we performed a wound healing assay in MCF7 cells, in which a line was formed by scratching the cell monolayer with a tip [33]. In this model the gap is mainly covered by cells that move to close it rather than cells that proliferate, at least at the early time points when cells do not have enough time to proliferate. At the 24 hour time point the result is a combination of proliferation and motility. The size of the gap was measured at different time points following stimulation using specialized software. Cells were treated with CRF at time 0 and were compared to vehicle only-treated cells at for the same period. Results are presented as % of the distance that remained open at that particular time point (Figure 3). Hence, at 12 hours 75.08 ± 1.57% of the initial gap was still open in control, vehicle treated cells, while 56.93 ± 1.17% of the gap was still open in CRF-treated cells. At 24 hours 55.42 ± 0.65% was still open in control cells while only 40.75 ± 0.35% of the gap was still uncovered in CRF-treated cells, suggesting that CRF promoted their motility. Given the fact that CRF reduced cell proliferation and apoptosis was not evident at 24 hours following stimulation (Figure 2), the results suggest that CRF stimulated motility that resulted in faster closure of the gap. The histograms represent the average of four independent experiments.

### 4. CRF induced MCF7 cell invasion through extracellular matrix

**Figure 4:**
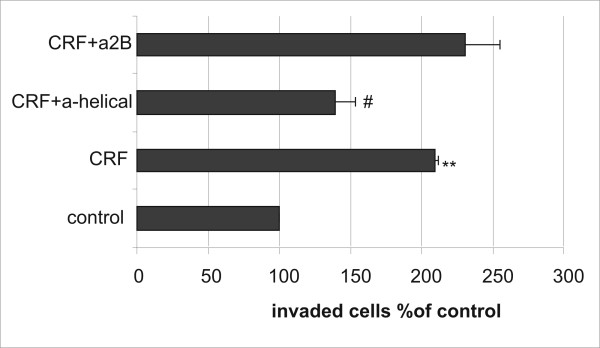
**CRF enhances the invasion of MCF7 through ECM via CRF1**. MCF7 cells were stimulated with 10^-8 ^M CRF or vehicle (control) and the invasion assay was performed according to the manufacturer's instructions. CRF augmented the invasion of the cells through the ECM. Pretreatment with the CRF1 antagonist (10^-6 ^M) abrogated the effect of CRF. Results represent the average of two independent experiments where each condition was assayed in triplicate. **p < 0.01 compared to untreated controls; #p < 0.05 compared to CRF-treated samples.

Invasion through the extracellular matrix (ECM) is a prerequisite for tumor metastasis. Since we found that CRF increased cell motility we further investigated whether it promoted invasiveness through extracellular matrix. MCF7 cells were plated on an ECM layer on a Boyden Chamber in the presence or absence of CRF and migration of cells through ECM was evaluated. As shown in Figure 4, incubation of MCF7 with CRF augmented the invasion of the cells through ECM. Moreover, the CRF1 antagonist, a-helical CRF abrogated the effect of CRF, while the CRF2 antagonist asstressin-2B had no effect (Figure 4).

### 5. CRF promoted actin cytoskeleton reorganization in MCF7 cells

**Figure 5:**
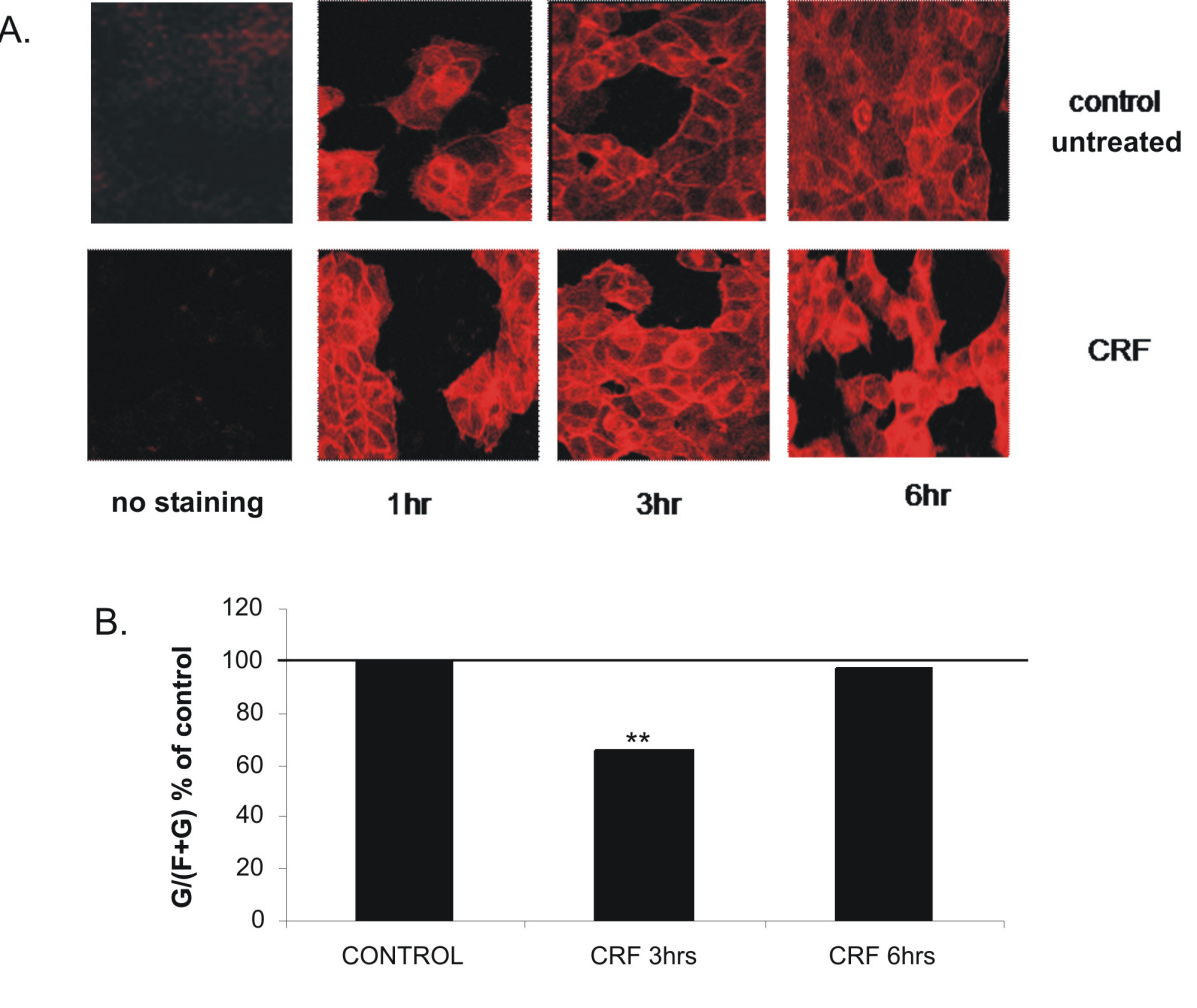
**A. CRF affects actin cytoskeleton**. MCF7 cells were plated in chamber-slides, stimulated with 10^-8 ^M CRF or vehicle (control) for 1, 3 and 6 hours and stained with rhodamine-phalloidine. Fluorescence was measured by confocal microscopy. Results are representative of three independent experiments.*B. CRF induces actin polymerization*. MCF7 cells were stimulated with CRF (10^-8 ^M) or vehicle (control) for 3 and 6 hours. Cells were harvested and the two fractions, soluble and insoluble, containing the monomeric (globular) and the polymerized (filamentous) actin respectively were isolated and analyzed by western blotting using antibody against actin. The relative proportion of F- and G-actin was determined by the concentration of monomeric (G) and total (F+G) actin and the result is presented as the G/(F+G) ratio induced by CRF compared to control; p < 0,01. Results represent the average of five independent experiments.

To determine the potential mechanism involved in CRF-induced motility and invasiveness we examined the effect of CRF on actin polymerization dynamics. Actin a major cytoskeletal component in eukaryotic cells occurs in two forms, the globular or G-actin, which polymerizes into the filamentous or F-actin. Filamentous actin is the major component of microfilaments, present in filipodia and lamellipodia, which are reported to facilitate cell migration [39]. In order to assess the role of CRF on cytoskeletal actin reorganization, we stained MCF7 cells stimulated with CRF or vehicle (0.1% acetic acid) for different time points with rhodamine-phalloidin that binds specifically to polymerized actin and visualized cells by confocal microscopy, evaluating actin filament structure and fluorescence intensity. As shown in Figure 5A, CRF induced alterations in actin cytoskeleton morphology, indicating changes in the polymerization dynamics of this protein. To quantify the extent of actin polymerization that occurred in the presence of CRF we analyzed the amount of monomeric G actin and compared it to the expression of total actin (monomeric G and polymeric F) providing the ratio between the two forms as previously reported [33,40,41]. Three hours following CRF stimulation the G/total actin ratio was significantly reduced, suggesting actin polymerization and formation of actin microfilaments (Figure 5B). Six hours later new monomeric actin was produced restoring the ratio of monomeric versus polymeric to the original state but with overall higher expression of actin, as indicated in Figure 5A.

**Figure 6:**
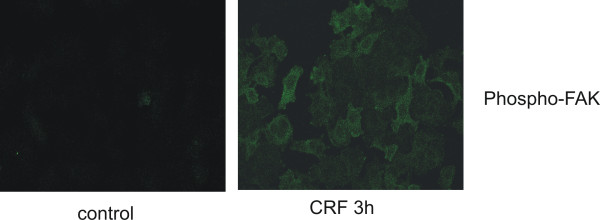
**CRF induces the phosphorylation of FAK**. MCF7 cells were treated with CRF (10^-8 ^M) or vehicle (control) for 3 hours, stained with phospho-FAK antibody and analyzed with confocal microscopy. Result is representative of three independent experiments.

FAK activation by phosphorylation is the first element, which may transmit extracellular signals to downstream signaling proteins, leading to actin reorganization [40,42-44] and is implicated in cell migration [33,41]. We, therefore, examined the effect of CRF on FAK phosphorylation in MCF7 cells. As shown in Figure 6, the phosphorylation of FAK was significantly increased in CRF treated MCF7 cells compared to vehicle-treated cells, indicating that it may also affect MCF7 cell invasiveness.

### 6. CRF increases prostaglandin production in MCF7 cells via Cox-1

**Figure 7:**
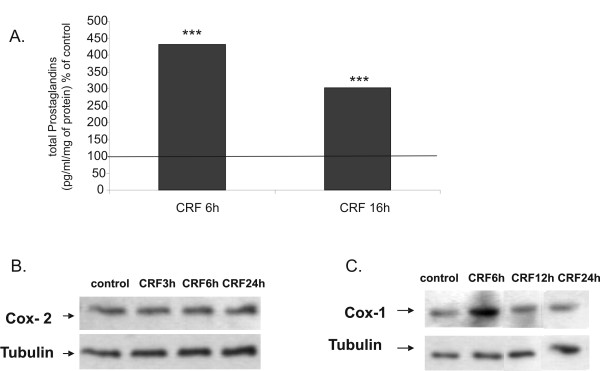
**CRF induces the expression of Cox-1 and the production of prostaglandins**. A. MCF7 cells were treated with CRF (10^-8 ^M) or vehicle (control) for 6 and16 hours and total prostaglandin production in the supernatant was measured by ELISA; ***p < 0.001; Results represent the average of three independent experiments. B. MCF7 cells were treated with CRF (10^-8 ^M) for 3, 6 and 24 hours and Cox-2 was measured by western blot. C. MCF7 cells were treated with CRF (10^-8 ^M) for 6, 12 and 24 hours and Cox-1 was measured by western blot. Results are representative of three independent experiments.

Cyclooxygenases (Cox), the enzymes that convert arachidonic acid into prostaglandins, have been causally linked to breast cancer cell proliferation, motility and invasiveness [45-48], thus the effect of CRF in prostaglandin production and Cox expression was investigated. We measured total prostaglandin production in supernatants of MCF7 cells stimulated with CRF by ELISA and found that CRF induced prostaglandin production in MCF7 cells (Figure 7A). CRF did not induce PGE2 production in MCF7 cells as measured by ELISA (data not shown). Indeed, COX-2 was not induced by CRF in this cell type (Figure 7B). In contrast, CRF induced COX-1 expression in a time-dependent manner, suggesting that COX-1 mediates CRF-induced prostaglandin production (Figure 7C).

## Discussion

Breast cancer growth is affected by several autocrine and paracrine factors that regulate tumor cell proliferation, apoptosis and metastatic potential. CRF is the major hypothalamic stress-induced neuropeptide but is also found in peripheral tissues. The aim of the study was to define the potential effect of CRF on breast cancer cell proliferation, apoptosis and metastatic potential.

We first investigated the expression of CRF receptors in MCF7 cells to confirm that the cells are responsive to CRF. In a previous report CRF1 receptor was detected in MCF7 cells [23]. In the present study we found that the CRF1a isoform was expressed in these cells and CRF2c was also present but at very low levels, indicating that the major mediator of CRF actions in MCF7 cells is CRF1 receptor. Indeed, inhibition of CRF2 receptors had no effect at least in the induction of cell invasion by CRF. These observations warrant further analysis of the CRF receptor system in primary breast cancer tissues that will support the significance of these receptors in breast cancer.

Earlier studies had shown that CRF suppressed breast cancer cell proliferation while it promoted proliferation in melanoma cells [6]. Our studies confirmed the suppressive effect of CRF on MCF7 proliferation. We further investigated the role of CRF on MCF7 cell apoptosis and found that CRF inhibited apoptosis. The effect of CRF on apoptosis varies depending on the cell type and the time detected. Thus, in PC12 rat pheochromocytoma cells CRF promoted apoptosis [28] while in neuroblastoma [29] and in melanoma cells it inhibited apoptosis [26]. An earlier study in MCF7 cells showed no effect of CRF on apoptosis using a less sensitive method, this of visualizing fragmented DNA [23]. Differences between cell types may be attributed to different factors that the cells may produce; i.e in Y79 neuroblastoma cells CRF inhibited caspase 3 activity [29], while PC12 cells undergo apoptosis in response to CRF due to production of FasL [28], which is not expressed in MCF7 cells.

The fact that CRF affected apoptosis and at the same time it inhibited cell proliferation may indicate changes in the cellular physiology that could contribute to a metastatic phenotype. Reduced cell proliferation, at least temporary, is required for cells to reorganize their cytoskeleton and promote motility. Indeed, CRF induced motility in MCF7 cells as demonstrated by a wound-healing assay. Cell motility is facilitated by cytoskeletal rearrangements that are characterized by actin polymerization. Our results indicated that CRF promoted polymerization of actin as determined by measuring the ratio of the monomeric versus the polymeric actin, as well as visualizing polymerized actin by immunofluorescence using confocal laser scanning microscopy. Increased actin polymerization is associated with dynamic changes in cytoskeletal structures that allow cells to migrate and metastasize [49]. Focal Adhesion Kinase is a cytoskeleton associated kinase that is activated by phosphorylation and mediates signals to promote cell adhesion and migration [50]. FAK also seems to play a role in tumor development since it has been shown that primary human cancer cells or cell lines overexpress the protein as well as its phosphorylated form [51,52]. In particular, FAK was found to overexpressed and to be highly activated in tumorigenic DU145 and PC-3 cells as well as in prostate cancer tissues from patients with metastasis [52,53] whereas in LNCaP cells that have a lower tumorigenic ability FAK was observed to be much lower [44,52]. We found that CRF promoted phosphorylation of FAK providing a potential mechanism for the actin reorganization and increased migration observed in response to CRF. It is, thus, likely that CRF initiates signals that promote cytoskeletal changes resulting in cell adhesion and migration by activating pathways that involve FAK phosphorylation.

The metastatic process requires that cells do not only have increased motility but they should also obtain the capacity to migrate through the ECM. For this purpose we examined the effect of CRF to promote invasion through ECM in MCF7 cells that have low metastatic potential. Indeed, treatment with CRF increased the invasiveness of MCF7 cells through ECM. Invasiveness, through ECM was measured using a boyden chamber assay, in which cells were plated on an ECM coated surface. Hence, cells should not only obtain the capability of migration, they should also be able to destroy the ECM in order to penetrate tissue barriers and metastasize. MCF7 breast cancer cells obtain this capability by expressing matrix metalloproteinases [54].

Cyclooxygenase activation and prostaglandin production has also been associated with increase in metastasis [47,55,56]. Inhibition of Cox-2 is associated with decrease in tumor growth and invasiveness [56,57]. Cox-1, an otherwise constitutively expressed Cox isoform, is also upregulated in breast cancer and is associated with increased prostaglandins and metastatic potential [56,58]. The primary Cox isoform expressed in MCF7 cells is Cox-1 [59]. We, therefore, examined the production of prostaglandins in response to CRF in MCF7 cells. CRF induced prostaglandin production but it did not alter PGE2 levels. In contrast, CRF increased the levels of Cox-1 suggesting that Cox-1-derived prostaglandins may mediate the effect of CRF on MCF7 cell invasiveness. Indeed, several reports have indicated that selective inhibition of Cox-1 results in inhibition of tumor growth and metastasis [56,58].

## Conclusion

In conclusion, CRF appears to positively affect tumor growth by inhibiting apoptosis and promoting cell migration and invasiveness. Our results provide a potential link between stress and tumor growth, suggesting that CRF secreted from autonomic neurons innervating peripheral tissues [25] may contribute to breast cancer metastasis. Given recent findings for the anti-tumor properties of CRF2 agonists [30] and the lack of CRF2 expression on breast cancer cells one may suggest that inhibition of CRF1 and activation of CRF2 may successfully inhibit tumor growth.

## Abbreviations

CRF: Corticotropin Releasing Factor; CRF1, CRF2: Corticotropin Releasing Factor receptor 1, or receptor 2; FAK: Focal Adhesion Kinase; UCN: Urocortin; COX: Cyclooxygenase; ECM: Extracellular Cell Matrix; PG: Prostaglandin; PGE2: Prostaglandin E2.

## Competing interests

The authors declare that they have no competing interests.

## Authors' contributions

AA participated in the molecular and cell biology studies and drafted the manuscript. ED participated in the immunofluorescence and confocal microscopy analyses and co-ordination of the study. MV participated in the analysis of CRF receptor expression, cell proliferation and apoptosis, data evaluation, co-ordination of the study and manuscript preparation. EK participated in the molecular and cell biology studies and data analysis. OR participated in the detection of CRF receptors. EA participated in the analysis of prostaglandin production and cyclooxygenase expression. CS and ANM participated in the design of the study and drafting of the manuscript. CT conceived of the study, participated in its design and drafted the manuscript. All authors read and approved the final manuscript.
